# 2-All­yloxy-5-nitro­benzoic acid

**DOI:** 10.1107/S1600536809024179

**Published:** 2009-06-27

**Authors:** Valquiria B. N. Ferreira, Haidi D. Fiedler, Faruk Nome, Adailton J. Bortoluzzi

**Affiliations:** a Depto. de Química–UFSC, 88040-900 Florianópolis, SC, Brazil

## Abstract

The mol­ecule of the title compound, C_10_H_9_NO_5_, is approximately planar, with the mean planes of the nitro, carboxyl and all­yloxy groups rotated by 8.1 (3), 7.9 (3) and 4.52 (18)°, respectively, from the plane of the benzene ring. Bond lengths in the aromatic ring are influenced by both electronic effects and strain induced by *ortho*-substitution. In the crystal structure, centrosymmetrically related mol­ecules are paired into dimers through strong O—H⋯O hydrogen bonds.

## Related literature

For information about chorismate mutase catalysis, see: Ziegler (1977 ▶); Castro (2004 ▶); Zhang *et al.* (2005 ▶). For related compounds, see: Ferreira *et al.* (2007 ▶); Jones *et al.* (1984 ▶). For the synthetic procedure, see: White *et al.* (1958 ▶). 
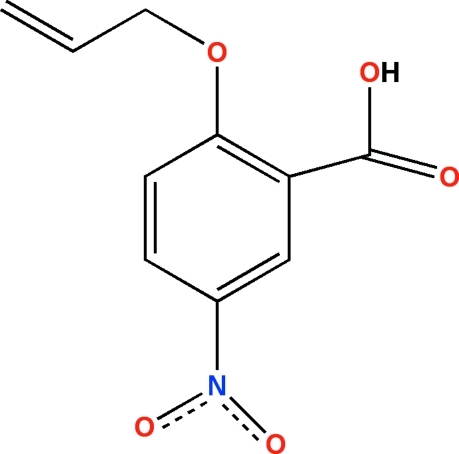


## Experimental

### 

#### Crystal data

C_10_H_9_NO_5_
*M* *_r_* = 223.18
Monoclinic, 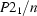

*a* = 3.9438 (6) Å
*b* = 9.0409 (7) Å
*c* = 28.804 (4) Å
β = 92.227 (11)°
*V* = 1026.2 (2) Å^3^
*Z* = 4
Mo *K*α radiation
μ = 0.12 mm^−1^
*T* = 293 K
0.50 × 0.40 × 0.26 mm

#### Data collection

Enraf–Nonius CAD-4 diffractometer
Absorption correction: none
2036 measured reflections
2000 independent reflections
1382 reflections with *I* > 2σ(*I*)
*R*_int_ = 0.023
3 standard reflections every 200 reflections intensity decay: 1%

#### Refinement

*R*[*F* ^2^ > 2σ(*F* ^2^)] = 0.046
*wR*(*F* ^2^) = 0.153
*S* = 1.06
2000 reflections
145 parameters
H-atom parameters constrained
Δρ_max_ = 0.28 e Å^−3^
Δρ_min_ = −0.19 e Å^−3^

### 

Data collection: *CAD-4 Software* (Enraf–Nonius, 1989 ▶); cell refinement: *SET4* in *CAD-4 Software*; data reduction: *HELENA* (Spek, 1996 ▶); program(s) used to solve structure: *SIR97* (Altomare *et al.*, 1999 ▶); program(s) used to refine structure: *SHELXL97* (Sheldrick, 2008 ▶); molecular graphics: *PLATON* (Spek, 2009 ▶) and *Mercury* (Macrae *et al.*, 2006 ▶); software used to prepare material for publication: *SHELXL97*.

## Supplementary Material

Crystal structure: contains datablocks global, I. DOI: 10.1107/S1600536809024179/rz2336sup1.cif

Structure factors: contains datablocks I. DOI: 10.1107/S1600536809024179/rz2336Isup2.hkl

Additional supplementary materials:  crystallographic information; 3D view; checkCIF report

## Figures and Tables

**Table 1:** Hydrogen-bond geometry (Å, °)

*D*—H⋯*A*	*D*—H	H⋯*A*	*D*⋯*A*	*D*—H⋯*A*
O22—H22⋯O21^i^	1.01	1.64	2.639 (2)	170

Symmetry code: (i) 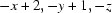.

**Fig. 1.**
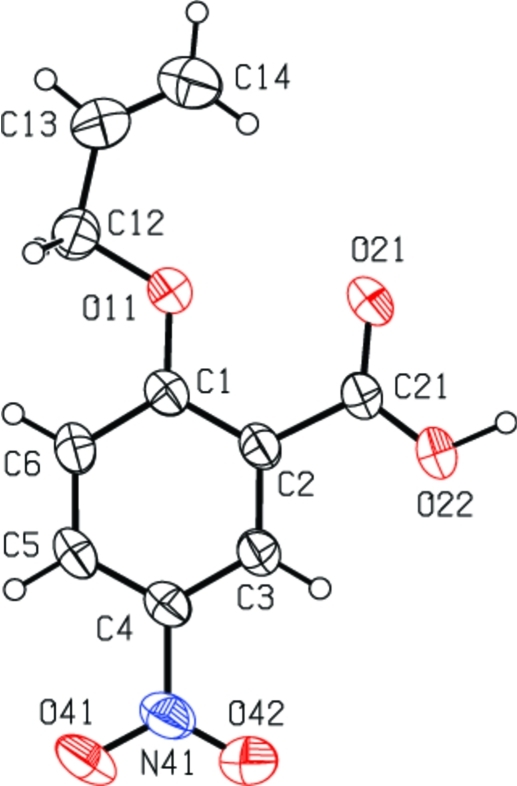
The molecular structure of the title compound with the labelling scheme. Displacement ellipsoids are shown at the 40% probability level.

**Fig. 2.**
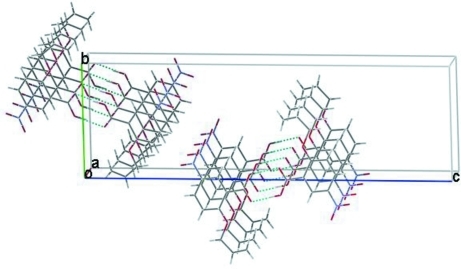
Partial packing diagram of the title compound viewed along the a axis. Intermolecular O—H···O hydrogen bonds are shown as dashed lines.

**Fig. 3.**
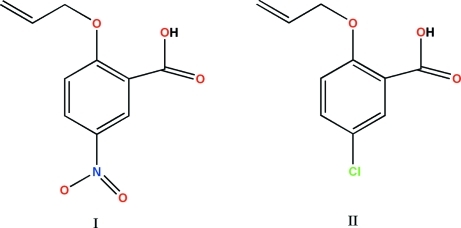
Schematic representations of the structures of (I) and (II).

## supplementary crystallographic information

### Comment

The unimolecular rearrangement of chorismate to prephenate, catalyzed by chorismate mutase, is a rare case of a [3,3] sigmatropic shift reaction in live organisms and it is a natural target in drug development, since corresponds to a key step in the pathway to form aromatic amino acids in plants, bacteria and fungi (Ziegler, 1977; Castro, 2004; Zhang *et al.*, 2005). Chorismate mutase catalyzes this intramolecular reaction without formation of an enzyme-substrate covalent intermediate and the proposed transition state structures in the gas phase, water and enzyme are characteristic of a concerted pericyclic rearrangement. Transition state stabilization seems to be responsible for only 10% of the enzymatic advantage over the water reaction (106-fold catalytic effect). Since we are interested in the systematic analysis of the influence of electrostatic stabilization and intramolecular hydrogen bonding in [3,3] sigmatropic Claisen rearrangements, a series of ethers derived from salicylic acid has been synthesized. The 2-allyloxy-5-nitrobenzoic acid (I, scheme 2) is a new synthesized compound and here we report its X-ray crystal structure.

A projection of the molecular structure and the numbering of the non-hydrogen atoms are shown in Fig. 1. Bond length data show that in the aromatic ring the C3—C4, C4—C5 and C5—C6 bonds are the strongest (shortest) C—C ring bonds, as a consequence of both electronic effects and strain induced by *ortho*-substitution at C1 and C2. The mean plane of nitro, carboxyl and allyloxy groups are deviated from the best plane of the phenyl ring by 8.1 (3)°, 7.9 (3)° and 44.52 (18)°, respectively. The electron withdrawing influence of the carboxyl group, combined with the strain introduced by the allyl ether, weakens the C1—C2 and C1—C6 bonds significantly and makes them longer than the other ring bonds. These effects in both bond lengths and coplanarity of the aromatic ring and the carboxyl group are similar to those found in the crystal structure of 2-allyloxy-5-chlorobenzoic acid (II) (Ferreira *et al.*, 2007). The nitro group in 1 has a small effect on the C3—C4—-C5 angle (121.5 (2)°), but the COOH group reduces the C1—C2—C3 angle from 120° (normal benzene ring) to 119.10 (18)°. The effect is practically identical to that found in compound (II, scheme 2), where the C3—C4—C5 angle is 120.4 (2)° and C1—C2—C3 angle is 119.37 (19)°. In both compounds, the observed effect evidently results from the presence of the allyloxy group in (I), lengthening both C1—C2 and C1—C6 bonds, and reducing the C2—C1—C6 angle to 119.09 (19)°. Closely similar effects are observed for 2-methoxymethoxybenzoic acid, where the *ortho*-substituent is electronically and sterically similar (Jones *et al.*, 1984).

In the carboxylic group, the C—O distances are very similar to each other. This indicates a high degree of delocalization of the π-electrons over the COOH backbone. Once the acid group is protonated, the similarity in bond lengths can be attributed to the very strong hydrogen bond (see Table 1). These intermolecular interactions also induce the formation of dimeric structures through center of symmetry. In the three-dimensional packing, the pairs of molecules are perfectly stacked along crystallographic *a* axis (Fig. 2).

### Experimental

Preparation of (I) followed closely the procedure described by White *et al.* (1958). A mixture of 9.15 g (0.05 mol) of 5-nitrosalycilic acid, 6.05 g (0.05 mol) of allyl bromide, 8.29 g (0.06 mol) of dry, powdered potassium carbonate, and sufficient dry acetone (about 30 ml) to give an easily stirred mass was stirred and refluxed for eight hours. Then the mixture was filtered, acidified with diluted acetic acid and the acetone removed by distillation under reduced pressure. The residue was initially obtained as an amorphous solid and yellow crystals of (I) were grown from aqueous acetone solution by slow evaporation at room temperature (m.p. 120–121°C).

### Refinement

All non-H atoms were refined with anisotropic displacement parameters. H atoms were placed at their idealized positions with distances of 0.93 and 0.97 Å and *U*_eq_ fixed at 1.2 times *U*_iso_ of the preceding atom for C—H_Ar_ and CH_2_, respectively. The H atom of the COOH group was found in a Fourier difference map and treated with riding model and its *U*_eq_ was also fixed at 1.2 times *U*_iso_ of the parent atom.

### Crystal data

**Table:**

C_10_H_9_NO_5_	*F*(000) = 464
*M**_r_* = 223.18	*D*_x_ = 1.445 Mg m^−^^3^
Monoclinic, *P*2_1_/*n*	Melting point = 393–394 K
Hall symbol: -P 2yn	Mo *K*α radiation, λ = 0.71073 Å
*a* = 3.9438 (6) Å	Cell parameters from 25 reflections
*b* = 9.0409 (7) Å	θ = 7.0–18.7°
*c* = 28.804 (4) Å	µ = 0.12 mm^−^^1^
β = 92.227 (11)°	*T* = 293 K
*V* = 1026.2 (2) Å^3^	Prismatic, yellow
*Z* = 4	0.50 × 0.40 × 0.26 mm

### Data collection

**Table:**

Enraf–Nonius CAD-4 diffractometer	*R*_int_ = 0.023
Radiation source: fine-focus sealed tube	θ_max_ = 26.0°, θ_min_ = 1.4°
graphite	*h* = −4→4
ω–2θ scans	*k* = −11→0
2036 measured reflections	*l* = −35→0
2000 independent reflections	3 standard reflections every 200 reflections
1382 reflections with *I* > 2σ(*I*)	intensity decay: 1%

### Refinement

**Table:**

Refinement on *F*^2^	Primary atom site location: structure-invariant direct methods
Least-squares matrix: full	Secondary atom site location: difference Fourier map
*R*[*F*^2^ > 2σ(*F*^2^)] = 0.046	Hydrogen site location: inferred from neighbouring sites
*wR*(*F*^2^) = 0.153	H-atom parameters constrained
*S* = 1.06	*w* = 1/[σ^2^(*F*_o_^2^) + (0.0699*P*)^2^ + 0.3672*P*] where *P* = (*F*_o_^2^ + 2*F*_c_^2^)/3
2000 reflections	(Δ/σ)_max_ < 0.001
145 parameters	Δρ_max_ = 0.28 e Å^−^^3^
0 restraints	Δρ_min_ = −0.19 e Å^−^^3^

### Fractional atomic coordinates and isotropic or equivalent isotropic displacement parameters (Å^2^)

**Table:**

	*x*	*y*	*z*	*U*_iso_*/*U*_eq_	
C1	0.8059 (5)	0.2710 (2)	0.13044 (7)	0.0456 (5)	
C2	0.7710 (5)	0.4115 (2)	0.10955 (7)	0.0459 (5)	
C3	0.6348 (5)	0.5267 (2)	0.13471 (7)	0.0489 (5)	
H3	0.6112	0.6200	0.1213	0.059*	
C4	0.5345 (5)	0.5033 (2)	0.17938 (7)	0.0490 (5)	
C5	0.5636 (5)	0.3662 (3)	0.20024 (7)	0.0528 (6)	
H5	0.4927	0.3520	0.2303	0.063*	
C6	0.6985 (5)	0.2510 (3)	0.17597 (7)	0.0514 (5)	
H6	0.7191	0.1584	0.1899	0.062*	
O11	0.9422 (4)	0.16211 (16)	0.10571 (5)	0.0563 (4)	
C12	0.9673 (6)	0.0159 (2)	0.12522 (8)	0.0573 (6)	
H12A	0.7446	−0.0186	0.1334	0.069*	
H12B	1.1133	0.0172	0.1531	0.069*	
C13	1.1101 (6)	−0.0839 (3)	0.09020 (10)	0.0689 (7)	
H13	1.1140	−0.1842	0.0974	0.083*	
C14	1.2299 (8)	−0.0467 (4)	0.05078 (11)	0.0840 (9)	
H14A	1.2321	0.0522	0.0419	0.101*	
H14B	1.3140	−0.1187	0.0313	0.101*	
C21	0.8708 (6)	0.4455 (2)	0.06121 (7)	0.0515 (5)	
O21	1.0274 (6)	0.3526 (2)	0.03810 (6)	0.0853 (7)	
O22	0.7886 (6)	0.5704 (2)	0.04562 (6)	0.0942 (8)	
H22	0.8322	0.5963	0.0124	0.113*	
N41	0.3876 (6)	0.6249 (3)	0.20513 (7)	0.0653 (6)	
O41	0.2631 (6)	0.5975 (2)	0.24230 (6)	0.0867 (6)	
O42	0.3925 (7)	0.7490 (3)	0.18876 (7)	0.1116 (9)	

### Atomic displacement parameters (Å^2^)

**Table:**

	*U*^11^	*U*^22^	*U*^33^	*U*^12^	*U*^13^	*U*^23^
C1	0.0446 (11)	0.0518 (12)	0.0407 (11)	−0.0036 (9)	0.0057 (8)	−0.0010 (9)
C2	0.0494 (11)	0.0512 (12)	0.0375 (10)	−0.0041 (9)	0.0088 (8)	0.0012 (9)
C3	0.0573 (12)	0.0506 (12)	0.0390 (11)	−0.0020 (10)	0.0061 (9)	0.0009 (9)
C4	0.0517 (12)	0.0564 (12)	0.0392 (11)	−0.0022 (10)	0.0065 (9)	−0.0054 (9)
C5	0.0577 (13)	0.0674 (14)	0.0340 (11)	−0.0063 (11)	0.0101 (9)	0.0005 (10)
C6	0.0580 (12)	0.0559 (13)	0.0408 (12)	−0.0042 (10)	0.0067 (9)	0.0078 (10)
O11	0.0705 (10)	0.0510 (9)	0.0486 (9)	0.0034 (7)	0.0170 (7)	0.0026 (7)
C12	0.0590 (13)	0.0529 (13)	0.0601 (14)	0.0016 (10)	0.0049 (11)	0.0068 (11)
C13	0.0679 (16)	0.0613 (15)	0.0774 (18)	0.0088 (12)	0.0018 (13)	−0.0051 (13)
C14	0.090 (2)	0.086 (2)	0.0776 (19)	0.0132 (16)	0.0175 (16)	−0.0120 (16)
C21	0.0664 (13)	0.0494 (12)	0.0396 (11)	−0.0019 (10)	0.0122 (10)	0.0035 (10)
O21	0.1322 (17)	0.0749 (12)	0.0518 (10)	0.0252 (11)	0.0437 (11)	0.0109 (9)
O22	0.166 (2)	0.0660 (12)	0.0536 (10)	0.0221 (12)	0.0488 (12)	0.0183 (9)
N41	0.0775 (14)	0.0721 (14)	0.0469 (11)	0.0071 (11)	0.0092 (10)	−0.0126 (10)
O41	0.1095 (15)	0.0985 (15)	0.0543 (11)	0.0051 (12)	0.0334 (10)	−0.0185 (10)
O42	0.192 (3)	0.0707 (13)	0.0742 (14)	0.0405 (15)	0.0377 (15)	0.0007 (11)

### Geometric parameters (Å, °)

**Table:**

C1—O11	1.340 (2)	C12—C13	1.481 (3)
C1—C6	1.405 (3)	C12—H12A	0.9700
C1—C2	1.410 (3)	C12—H12B	0.9700
C2—C3	1.389 (3)	C13—C14	1.291 (4)
C2—C21	1.493 (3)	C13—H13	0.9300
C3—C4	1.377 (3)	C14—H14A	0.9300
C3—H3	0.9300	C14—H14B	0.9300
C4—C5	1.380 (3)	C21—O21	1.250 (3)
C4—N41	1.459 (3)	C21—O22	1.253 (3)
C5—C6	1.373 (3)	O22—H22	1.0056
C5—H5	0.9300	N41—O42	1.217 (3)
C6—H6	0.9300	N41—O41	1.221 (3)
O11—C12	1.438 (3)		
			
O11—C1—C6	122.9 (2)	O11—C12—C13	108.42 (19)
O11—C1—C2	118.00 (17)	O11—C12—H12A	110.0
C6—C1—C2	119.09 (19)	C13—C12—H12A	110.0
C3—C2—C1	119.10 (18)	O11—C12—H12B	110.0
C3—C2—C21	116.95 (19)	C13—C12—H12B	110.0
C1—C2—C21	123.95 (18)	H12A—C12—H12B	108.4
C4—C3—C2	120.2 (2)	C14—C13—C12	127.0 (3)
C4—C3—H3	119.9	C14—C13—H13	116.5
C2—C3—H3	119.9	C12—C13—H13	116.5
C3—C4—C5	121.5 (2)	C13—C14—H14A	120.0
C3—C4—N41	119.5 (2)	C13—C14—H14B	120.0
C5—C4—N41	118.92 (19)	H14A—C14—H14B	120.0
C6—C5—C4	119.12 (18)	O21—C21—O22	122.7 (2)
C6—C5—H5	120.4	O21—C21—C2	120.8 (2)
C4—C5—H5	120.4	O22—C21—C2	116.47 (19)
C5—C6—C1	121.0 (2)	C21—O22—H22	120.0
C5—C6—H6	119.5	O42—N41—O41	122.7 (2)
C1—C6—H6	119.5	O42—N41—C4	119.0 (2)
C1—O11—C12	119.37 (16)	O41—N41—C4	118.3 (2)

### Hydrogen-bond geometry (Å, °)

**Table:**

*D*—H···*A*	*D*—H	H···*A*	*D*···*A*	*D*—H···*A*
O22—H22···O21^i^	1.01	1.64	2.639 (2)	170

Symmetry codes: (i) −*x*+2, −*y*+1, −*z*.
